# Supplementary material for: Bacterial cheating drives the population dynamics of cooperative antibiotic resistance plasmids
Source: Mol Syst Biol. 2013 Aug 6;9:683. doi: 10.1038/msb.2013.39 (PMC3779801; doi:10.1038/msb.2013.39)
Supplement: Supplementary Information [file msb201339-s1.pdf]

Supporting Information for

Bacterial Cheating Drives the Population Dynamics of Cooperative Antibiotic Resistance Plasmids

Eugene A. Yurtsev, Hui Xiao Chao, Manoshi Sen Datta, Tatiana Artemova and Jeff Gore

Supplementary Figures

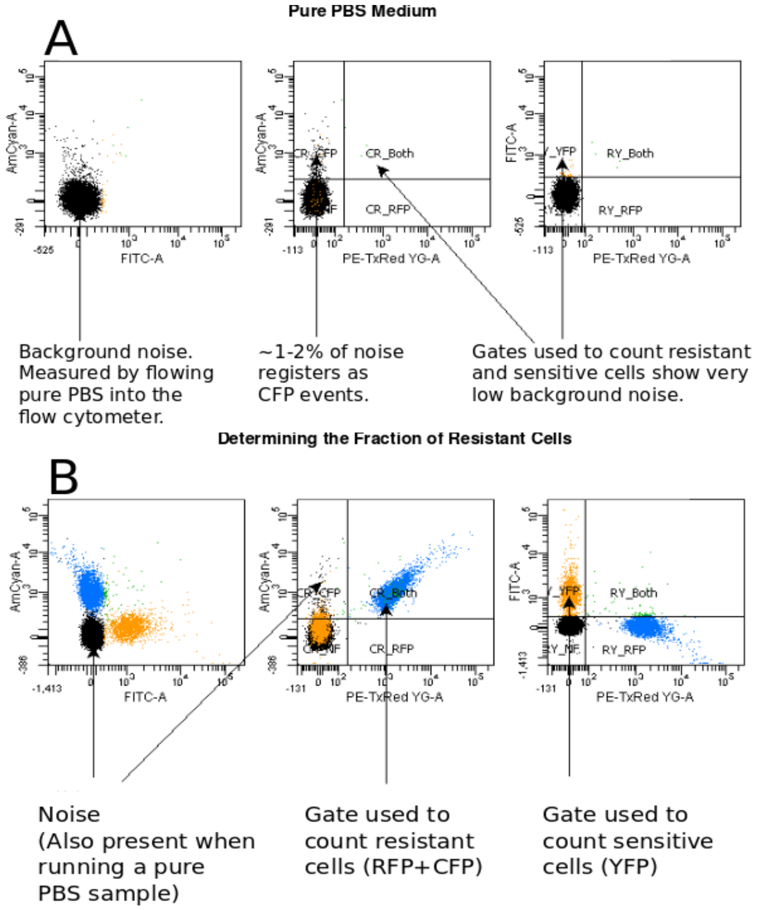

Supplementary Figure 1: Measurement of resistant fraction using flow cytometry. Shown is raw data from the flow cytometer: (**A**) PBS medium with no cells, and (**B**) a mixture of resistant and sensitive cells in PBS. To calculate the fraction of resistant bacteria, the number of fluorescent events registered in the gates corresponding to resistant and sensitive cells is counted (**B**). In addition, we measure the rate of false positives due to noise (**A**), and use it to correct the fraction of resistant bacteria. Because the noise level is low, the difference between corrected and uncorrected fractions is only ~0.01-0.03. The error in the fraction due to binomial counting statistics is small (~0.01) since there are thousands of events.

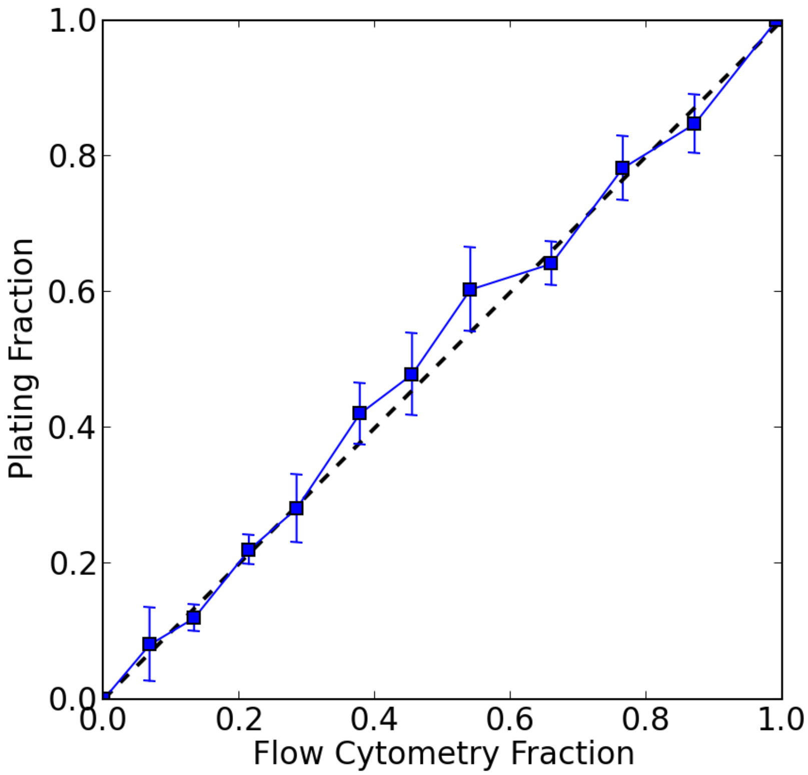

Supplementary Figure 2: Calibration of resistant fraction. The fraction of resistant cells measured by plating is equivalent to that measured on the flow cytometer. Sensitive and resistant cultures were grown from single colonies in 5 mL of LB with antibiotics at 37ºC for 23 hours. The saturated sensitive and resistant cultures were mixed at different ratios yielding 12 mixed cultures. For each mixed culture, the fraction of resistant cells was determined using flow cytometry (Supplementary Figure 2). In addition, each mixed culture was plated on an LB-agar Petri dish, and the fraction of resistant cells was determined by counting the number of resistant and sensitive colony forming units (CFUs). Resistant and sensitive cells had different fluorescent markers, and were easy to tell apart using a fluorescent microscope. Error bars represent binomial counting error. The error associated with the fraction determined using flow cytometry is smaller than the size of the symbol.

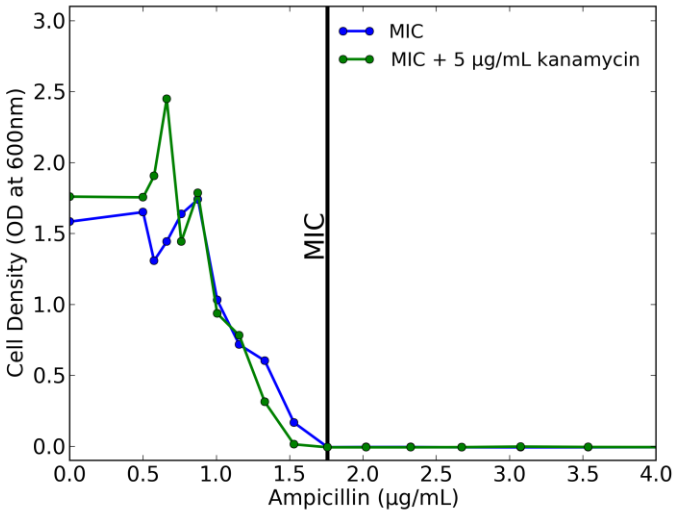

Supplementary Figure 3: Growth of sensitive cells in the antibiotic. The minimum inhibitory concentration (MIC) was determined by the lowest ampicillin concentration that inhibited bacterial growth (i.e., yielding no visible cell growth). In the experiment shown above, this concentration corresponds to ~1.8 μg/mL, and was not affected by the addition of 5 μg/mL of kanamycin. Notably, ampicillin starts to affect the growth of sensitive cells even at concentrations below the MIC. This fact is ignored by our model, which implies that the model's MIC may be slightly different from the measured MIC. To measure the MIC, sensitive cells were grown in 5 mL of LB and 5 μg/mL of kanamycin for 23 hours. The saturated culture was then diluted into media containing different ampicillin concentrations and grown for 23 hours starting at an initial cell density of ~$4 \cdot 10^3$ CFU/mL. The 23 hour growth cycle was used to closely mimic the competition experiments. Our MIC was measured at a low cell density to more accurately characterize the effect of antibiotic on killing single cells. Alternatively, measuring the MIC using the standard cell density of ~$5 \cdot 10^5$ CFU/mL and 20 hours of growth, yielded an MIC of ~2 μg/mL. Regardless of the method used to measure the MIC, ampicillin starts to affect the growth of sensitive cells even at concentrations below the MIC.

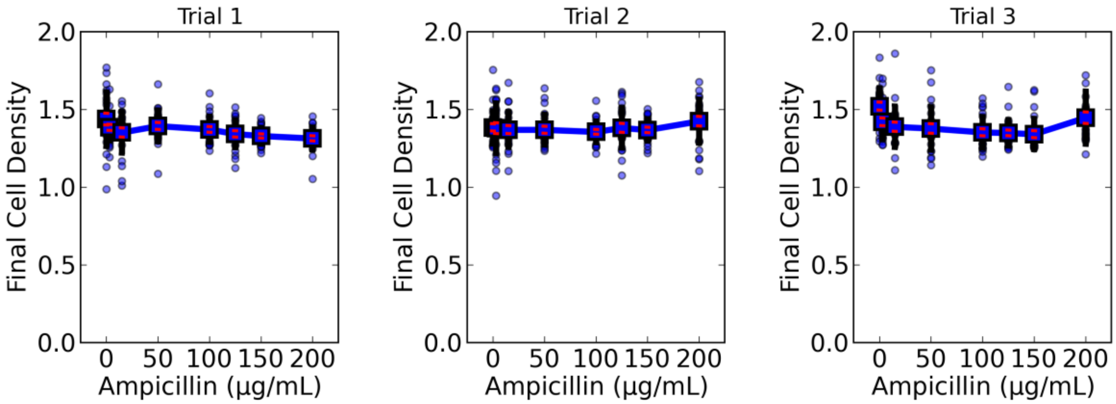

Supplementary Figure 4: Final cell density is independent of ampicillin concentration. Shown is the cell density after 23 hours of growth in LB with 5 μg/mL of kanamycin and varying concentrations of ampicillin. Experiments were carried out at a dilution factor of 100x. Circles correspond to individual data points, squares to the means, the black error bars to the standard deviations, and the red error bars to the standard errors of the mean. Cell density corresponds to the optical density measured at 600 nm. Experiments were repeated on 3 different days (trials 1-3). These are the three experiments from which Figure 3 in the main text was generated.

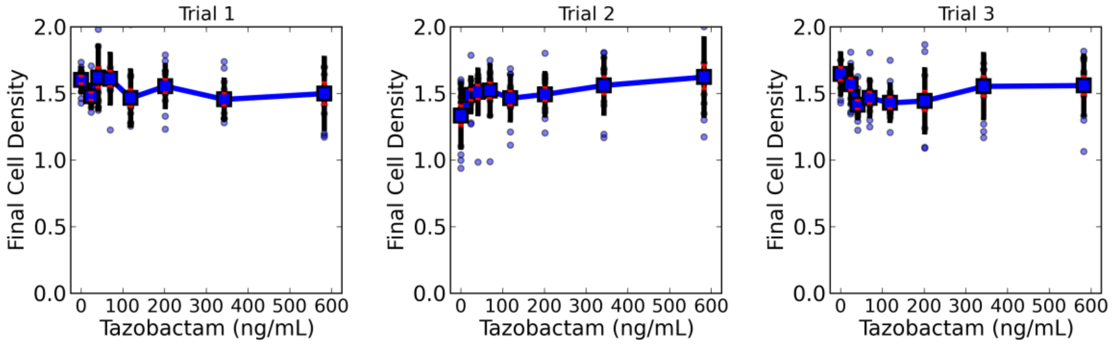

Supplementary Figure 5: Final cell density is independent of tazobactam concentration. Shown is the cell density after 23 hours of growth in LB with 5 µg/mL of kanamycin, 2 µg/mL of ampicillin and varying concentrations of tazobactam. Experiments were carried out at a dilution factor of 100x. Circles correspond to individual data points, squares to the means, the black error bars to the standard deviations, and the red error bars to the standard errors of the mean. Cell density corresponds to the optical density measured at 600 nm. Experiments were repeated on 3 different days (trials 1-3). These correspond to the three experiments from which Figures 4B, C in the main text were generated.

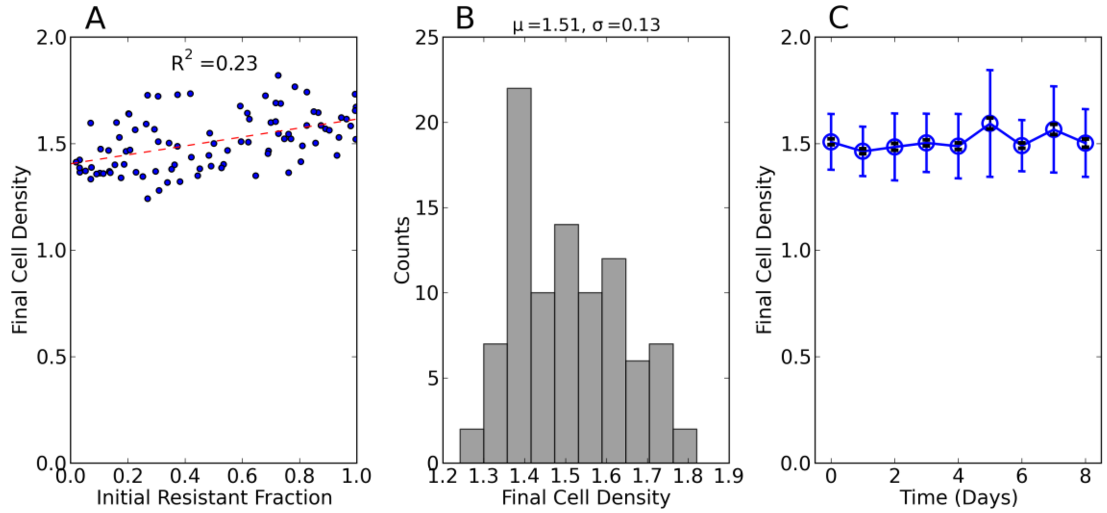

Supplementary Figure 6: Final cell density vs initial fraction and time. Cells were grown for 23 hours in LB with 5 µg/mL of kanamycin, and 100 µg/mL of ampicillin. (A) Shows the dependence of the final cell density (OD_{600}) on the initial resistant fraction at the end of the first day. The final cell density depends weakly on the initial resistant fraction. (B) A histogram of the final cell density at the end of the first day. (C) The saturated cultures were propagated at a dilution factor of 100x over multiple days. The final cell density does not change significantly with time. The blue error bars represent the standard deviation while the black error bars represent the standard error of the mean. Final cell density corresponds to the optical density measured at 600 nm. Data comes from the same experiment as used to generate Figure 1A in the main text.

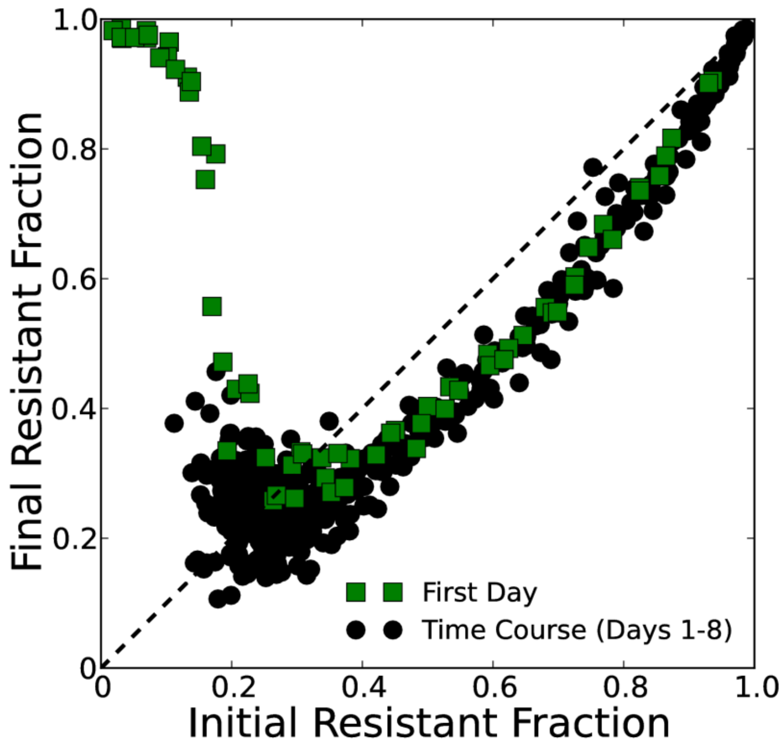

Supplementary Figure 7: Difference equations obtained on consecutive days. The difference equation obtained on the first day (green squares) slightly overestimates the resistant fraction seen on consecutive days (black circles). Cultures were grown in LB with 5 µg/mL kanamycin, 100 µg/mL ampicillin at a dilution factor of 100x.

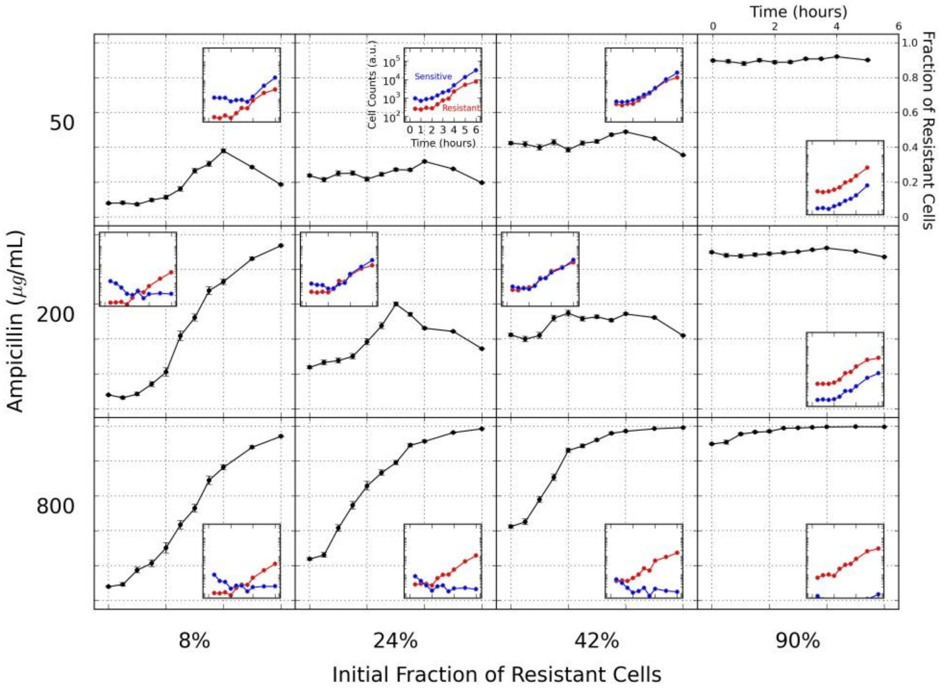

Supplementary Figure 8: Intra-day growth dynamics in ampicillin of bacterial populations containing both resistant and sensitive cells. Bacterial populations containing resistant and sensitive cells were grown in 200 µL of LB with specified concentrations of ampicillin. Every 30 to 60 minutes, 1 µL of each bacterial culture was transferred into 199 µL of PBS, and the sample was measured on the flow cytometer. Each measurement yielded the number of resistant and sensitive cells (red and blue lines in the insets), as well as the fraction of resistant cells (black lines) during one time point in the course of bacterial growth in the antibiotic.

The data shows that at lower initial antibiotic concentrations and higher initial fractions of resistant cells, sensitive cells grow virtually unhindered by the antibiotic. In contrast, at higher antibiotic concentrations and lower initial fractions of resistant cells, the antibiotic can kill a significant fraction of the sensitive cells before it is inactivated. In addition, consistent with our

modeling assumptions, sensitive cells can recover and resume growth after experiencing cell death.

The measurement at time $t = 0$ was carried at a higher cell density on the flow cytometer (different dilution into PBS), yielding cell counts that were artificially higher than the cell counts for $t > 0$. Consequently, we omitted the cell counts for $t=0$, but we did include the fraction of resistant cells since it is unaffected by the different cell density.

The error bars on the fraction of resistant cells show the error associated with small cell numbers (approximated as ~sqrt(f(1-f)/N), where f is the fraction of resistant cells and N is the total number of cells). This error is less than 5% for most measurements (since N > 100 cells).

When measuring the fraction of resistant cells continuously over the course of bacterial growth, rather than just at the initial and final times as is done elsewhere in this study, one must worry about additional sources of noise. One issue is that the fluorescence of sensitive cells decreases during the first few hours of growth, leading to overestimation of the fraction of resistant cells. In addition, it is possible that sensitive cells can still lyse in PBS if they were on the verge of lysing before being diluted into PBS. This means that the measured fraction of resistant cells may exhibit some dependence on the time elapsed between dilution into PBS and the subsequent measurement on the flow cytometer. Re-measurements of the same samples in PBS gave mostly the same fractions of resistant cells (i.e., within the shown error bars); however, a few of the fractions were off by ~10-15%. Nonetheless, most of the dynamics are dominated by significantly larger changes in fraction than can be caused by noise.

Cultures were grown at 37ºC in a shaking incubator in the absence of kanamycin.

Also, please see the discussion in Supplementary Figure 24 regarding the change in the equilibrium fractions.

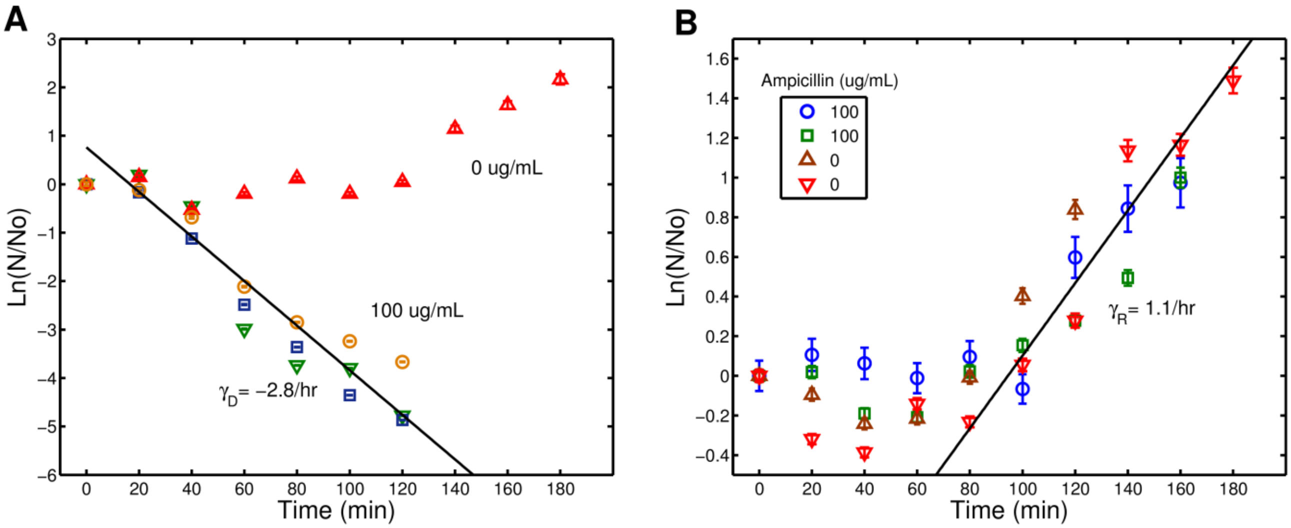

Supplementary Figure 9: Growth dynamics in ampicillin of sensitive cells and resistant cells grown separately. Resistant and sensitive cells were grown at 37ºC in 5 mL of LB and 5 µg/mL of kanamycin with either 0 µg/mL or 100 µg/mL of ampicillin. Every 20 minutes, the number of Colony Forming Units (CFUs), N, was obtained by plating cultures on LB-agar Petri dishes. (A) Sensitive cells exhibit a lag phase ~30-120 minutes, and die at a rate of ~2.8/hr when ampicillin is present. (B) Resistant cells exhibit a lag phase of ~80-100 minutes, and grow at a rate of ~ 1.1/hr, regardless if grown either in the absence or presence of ampicillin. In modeling growth in the antibiotic, we took the lag time of both resistant and sensitive cells to be 1 hour long. The different markers correspond to different trials. *No* represents the number of CFUs at time 0 in each trial.

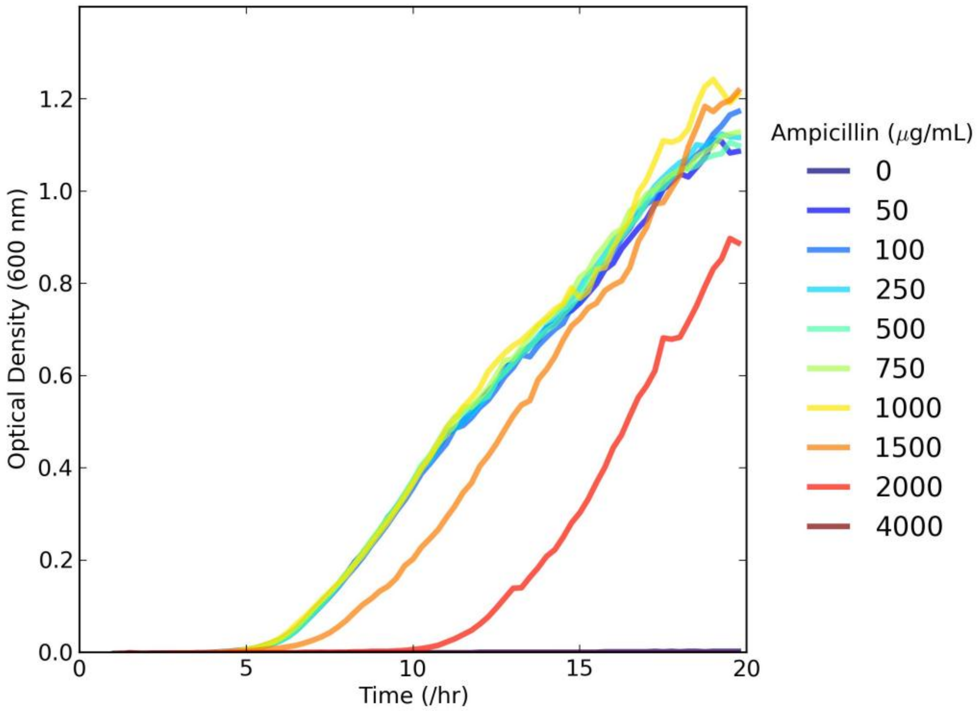

Supplementary Figure 10: Resistant cells in ampicillin. The growth of resistant cells is unaffected at the range of antibiotic concentrations probed in our experiments (up to ~200 µg/mL). Resistant cells were inoculated at an initial cell density corresponding to a dilution of ~1:10^5 from saturation into a 96-well plate with 200 µL of LB supplemented with ampicillin. The plate was placed into a shaking incubator at 37ºC, with optical density measurements taken every ~15 minutes. This strain is equivalent to the one used in the main text except for its kanamycin resistance plasmid which encodes a yellow fluorescent protein instead of a cyan fluorescent protein.

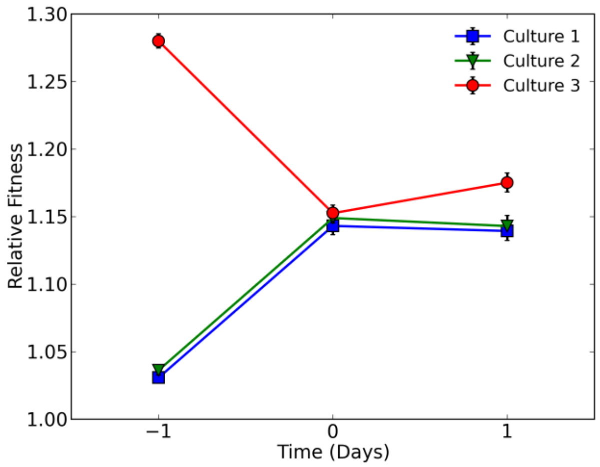

Supplementary Figure 11: Sensitive cells grow faster than resistant cells in the absence of the antibiotic ampicillin.

3 sensitive and 3 resistant cultures were grown from single colonies in 5 mL of LB with antibiotics at 37ºC for 23 hours. Each of the three saturated sensitive culture was mixed with one of the three saturated resistant cultures. The fraction of resistant cells in the mixed cultures was measured using flow cytometry (Day -1). The mixed cultures were then diluted by a factor of 100x into a 96-well plate in which they were grown in 200 µL of LB with 5 µg/mL kanamycin for a day. The fraction of resistant cells in the saturated cultures grown in the 96-well plates was measured using flow cytometry (Day 0). Cultures were propagated for another day (Day 1) under the same growth conditions as in Day 0 (diluted by factor of 100x into 96-well plate with 200 µL of LB at 5 µg/mL kanamycin), and the fractions of resistant cells were measured again using flow cytometry.

The relative fitness between the sensitive and resistant cells was calculated for each day of growth using the equation $r = \ln(N_{Sf}/N_{Si})/\ln(N_{Rf}/N_{Ri})$, where N_{Si}, N_{Sf}, N_{Ri}, N_{Rf} are the initial sensitive cell density, final sensitive cell density, initial resistant cell density, and final resistant cell density, respectively. The densities of the subpopulations were determined by combining flow cytometry measurements (yielding fractions of each subpopulation) together with optical

density measurements at 600 nm (yielding total density of bacterial population). The initial densities correspond to those of the freshly inoculated culture, whereas the final densities correspond to those of the saturated culture (after a day of growth). For each culture, error bars in the relative fitness represent the standard error of the mean over multiple replicates.

Importantly, we found that the relative fitness on Day -1 is an unreliable measure of the relative fitness between the two strains. Prior to being co-cultured together on Day -1, the two strains were grown separately in 5 mL of LB. We suspect that random differences between the growth histories of the two strains when grown separately lead to fluctuations in the relative fitness measured on Day -1.

For the following days (Days 0 and 1), the two strains experienced the same growth histories, and the measurements of the relative fitness across different cultures yielded consistent results. Consequently, all experiments were carried out after co-culturing the two strains together for a day in the absence of ampicillin before using them to measure difference equation maps.

The value of the relative fitness, r, used for modeling was the average of the relative fitness values across the three cultures obtained on Day 1 (r=1.15).

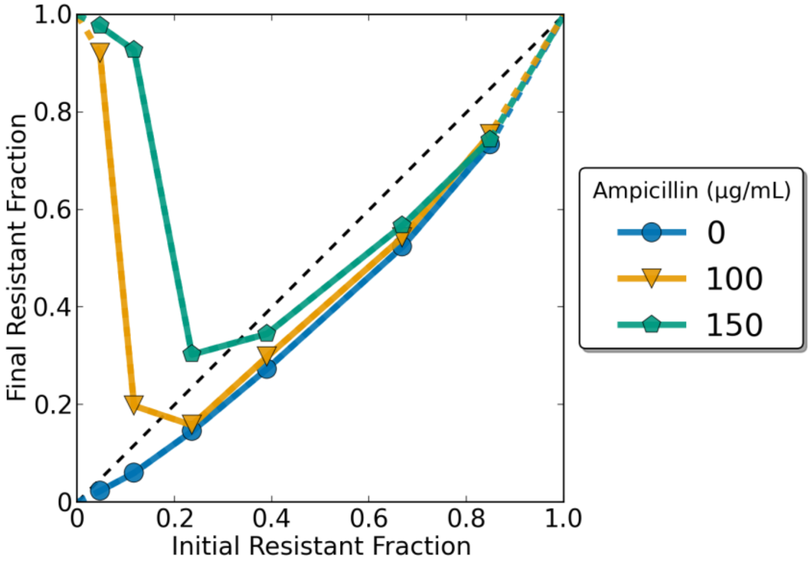

Supplementary Figure 12: Difference equation with a different plasmid. The evolutionary dynamics are not specific to the type of plasmid that encodes the β-lactamase gene. Shown are difference equations that were mapped for a resistant strain in which the resistance was carried by the pBR322 plasmid (New England Biolabs, Beverly, MA, identical to TEMwt). Unlike the resistance plasmid used in the main text, this plasmid does not encode a fluorescent protein. Quantitative differences are expected as hydrolysis rates, growth rates, lag times may vary between different plasmids; however, the qualitative features of the evolutionary dynamics remain robust; i.e., phenotypes have high fitness when rare, and for every ampicillin concentration, there is a characteristic fraction of resistant cells at equilibrium. Data was acquired in LB with 5 µg/mL of kanamycin at 100x dilution.

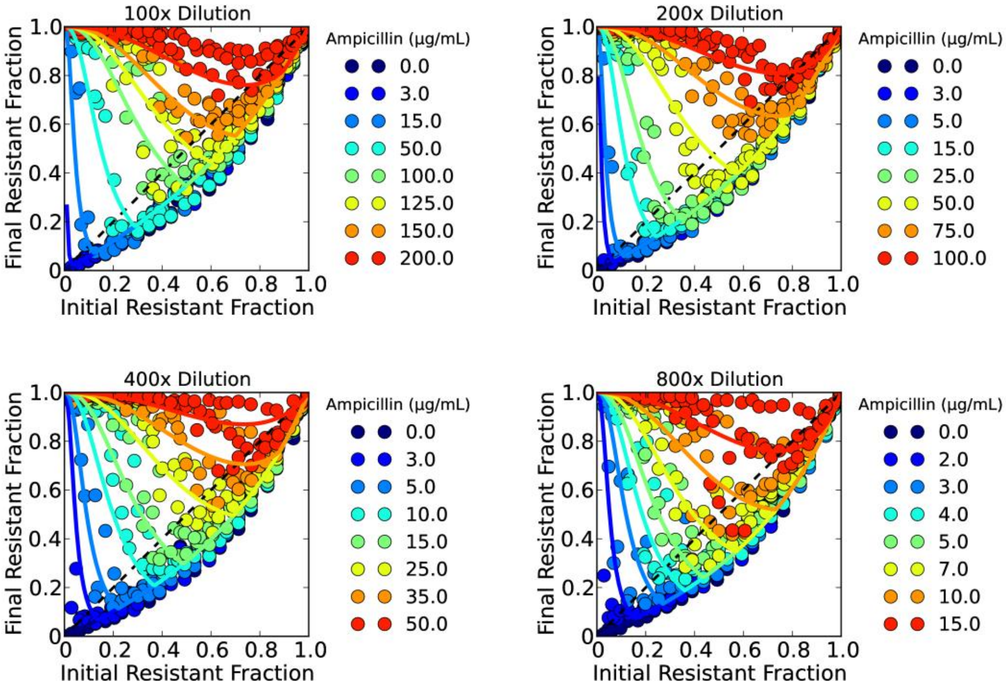

Supplementary Figure 13: Difference equation maps at different dilution factors. Each difference equation was measured on 3 different days. The 100x and 200x difference equations shown in the main text (Figure 3A, B) only include a subset of the data. Solid curves represent a single fit of the model to all experimental data.

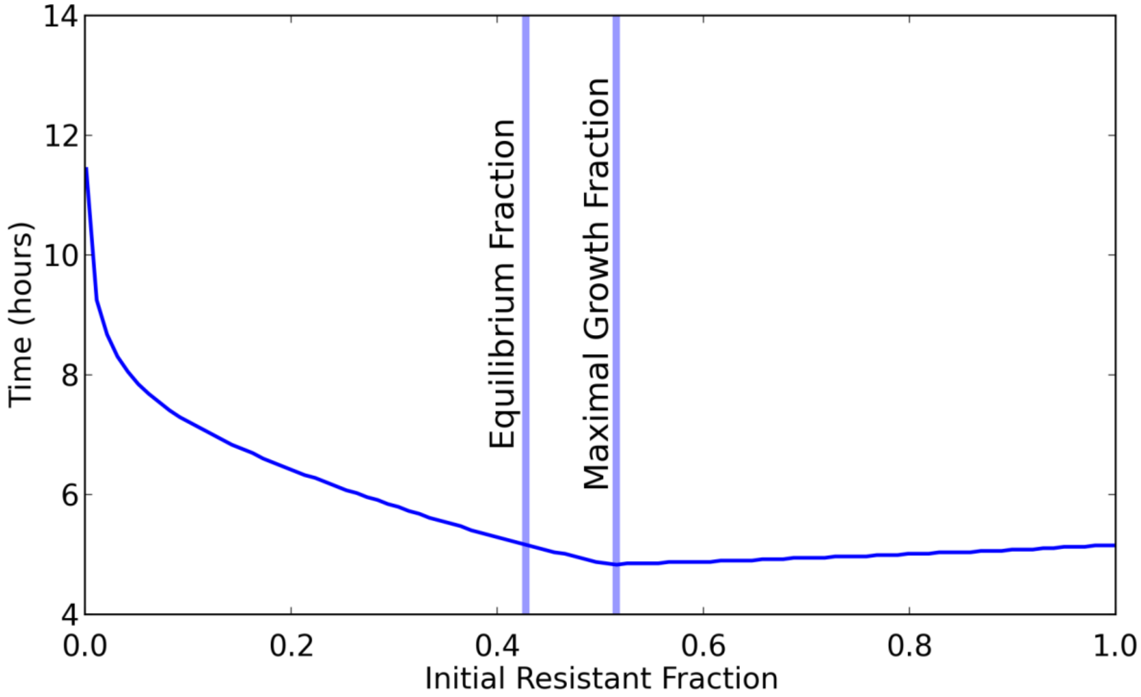

Supplementary Figure 14: A fraction of resistant and sensitive cells maximizes population growth rate. Simulations indicate that the fraction that maximizes the population growth rate (the time to reach saturating cell density) corresponds neither to the equilibrium fraction nor to an entirely resistant population. Notably, at the equilibrium fraction, the population growth rate is not substantially lower than that of a purely resistant population. This simulation was carried for the 100 μg/mL ampicillin and 100x dilution factor condition.

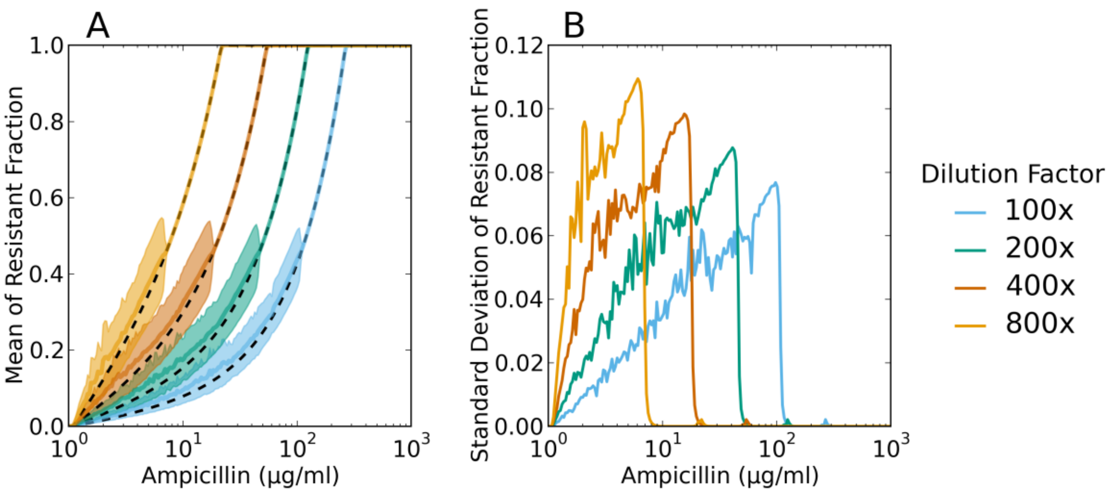

Supplementary Figure 15: Oscillatory dynamics. Equilibrium points can be stable or unstable. As the antibiotic concentration decreases, the slope of the difference equation at the fixed point increases in magnitude, and the equilibrium points become unstable. For an unstable equilibrium point, the resistant fraction starts to oscillate about that fixed point. Shown are the results of simulations of the evolutionary dynamics over the course of 50 days. For each antibiotic concentration and dilution factor, the population was started at an initial resistant fraction slightly offset from the equilibrium fraction, and the first 10 days of simulation were disregarded. (A) Shown are the mean resistant fraction (dark thick line, color), the standard deviation of the resistant fraction (lighter color) and the location of the fixed point (black dashed line). Note that for unstable fixed points, the mean fraction is slightly higher than the equilibrium fraction due to the asymmetric nature of the oscillations. (B) Shown is the standard deviation as a function of the ampicillin concentration. The size of the fluctuations is generally small (standard deviation lower than 0.1), making it difficult to tell apart the oscillations from experimental noise in this system. The parameters used in the simulations were acquired from fits to the experimental data (described in the main text). Parameter values are provided in the modeling

section of the Supplementary Materials.

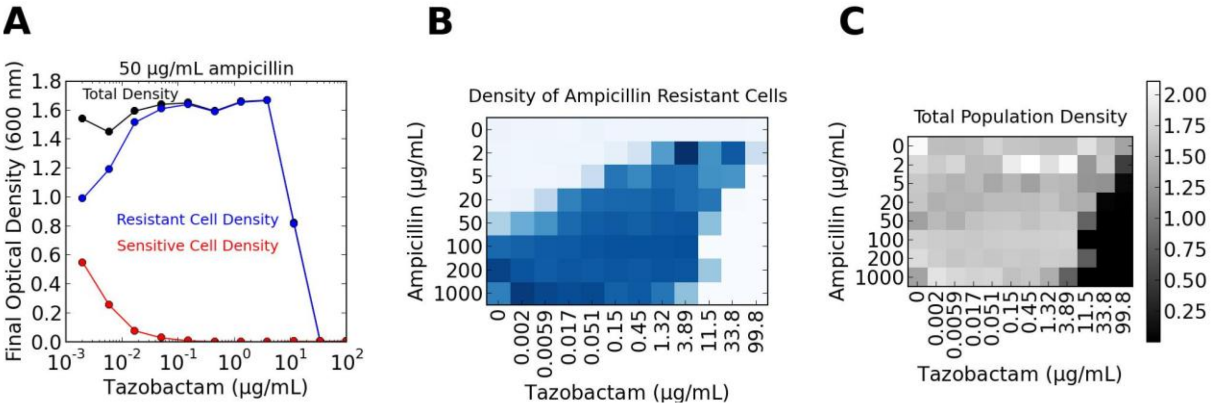

Supplementary Figure 16: Selection for resistance in ampicillin and tazobactam. (A) While high concentrations of the inhibitor successfully kill the bacterial population, low and intermediate concentrations of the inhibitor accelerate the spread of resistant cells. Heat maps showing (B) the final density of resistant subpopulation (blue is high density) and (C) the final density of entire bacterial population (white is high density) as a function of the antibiotic and inhibitor concentrations. Subplot (A) corresponds to a slice of subplots (B) and (C) subplots at a fixed ampicillin concentration of 50 µg/mL. In these experiments, a saturated culture with ~10% resistant cells was diluted by a factor 100x into a medium of LB supplemented with ampicillin and tazobactam. After 23 hours of growth, the density of resistant and sensitive cells was measured by combining flow cytometry measurements with an optical density measurement at 600 nm. No kanamycin was present in these experiments.

Also, please see the discussion in Supplementary Figure 24 regarding the change in the equilibrium fractions.

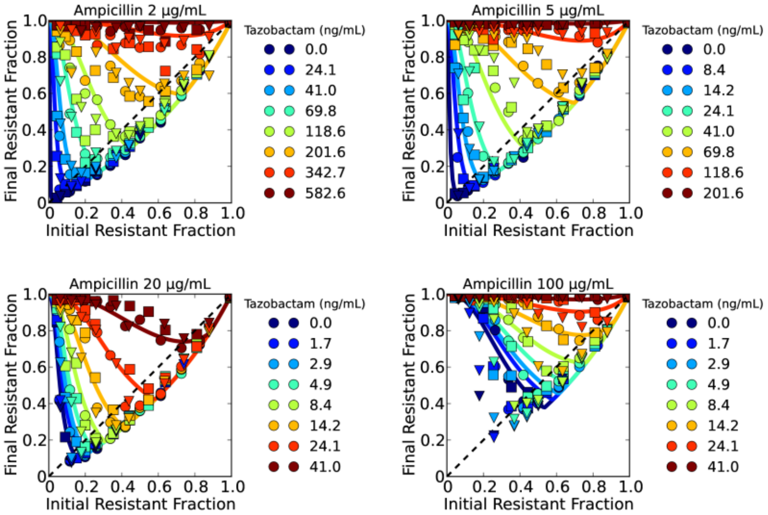

Supplementary Figure 17: Difference equation maps in the presence of the inhibitor tazobactam. Each difference equation was measured on 3 different days. The data and analysis presented in the main text in Figures 4B, 4C use these difference equation maps. Data was collected at a dilution factor of 100x. Solid curves represent a single fit of the model to all experimental data.

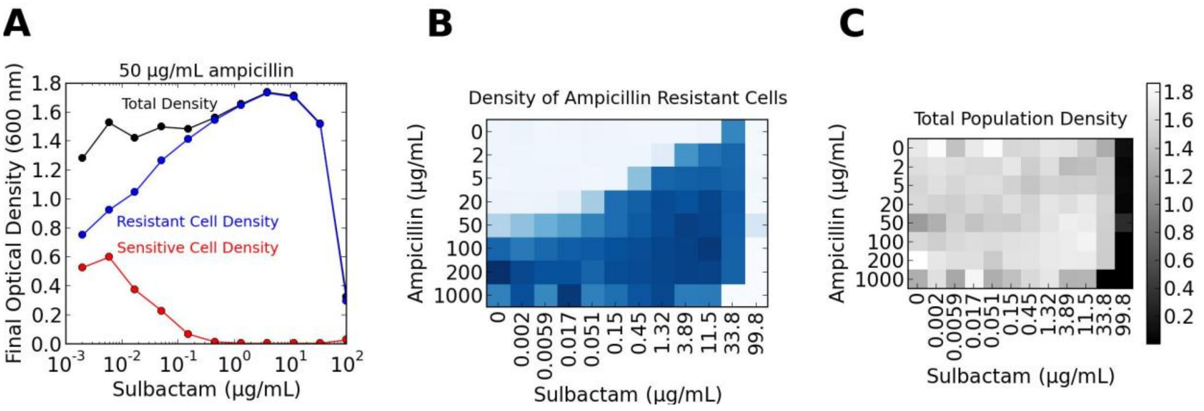

Supplementary Figure 18: Selection for resistance may be possible at clinically relevant concentrations. Clinically, ampicillin is combined often with the beta-lactamase inhibitor sulbactam. Depending on how the drugs are administered, the peak serum concentration of ampicillin may be between 40 µg/mL – 150 µg/mL while that of sulbactam may be between 10 µg/mL – 120 µg/mL (Foulds, 1986; Foulds *et al*, 1983; Bush, 1988). (A-C) Resistant cells spread at an accelerated rate in the lower concentration ranges of ampicillin/sulbactam while at the higher concentration ranges the growth of the bacterial population is inhibited.

Although resistant cells proliferate across a range of clinically relevant concentrations in our experiments, we want to stress that the minimum inhibitory concentrations of ampicillin/sulbactam is expected to change with the microorganism, and with the initial cell density of the bacterial population. Moreover, the use of ampicillin together with sulbactam is clinically known to be effective against many bacterial infections.

Subplot (B) shows a heat map of the final density of resistant subpopulation (blue is high density). Subplot (C) shows a heat map of the final density of entire bacterial population (white is high density) as a function of the antibiotic and inhibitor concentrations. Subplot (A) corresponds to a slice of subplots (B) and (C) at an ampicillin concentration of 50 µg/mL.

In these experiments, a saturated culture with ~10% resistant cells was diluted by a factor 100x into a medium of LB supplemented with ampicillin and sulbactam. After 23 hours of growth, the density of resistant and sensitive cells was measured by combining flow cytometry measurements with an optical density measurement at 600 nm. No kanamycin was present in these experiments.

Also, please see the discussion in Supplementary Figure 24 regarding the change in the equilibrium fractions.

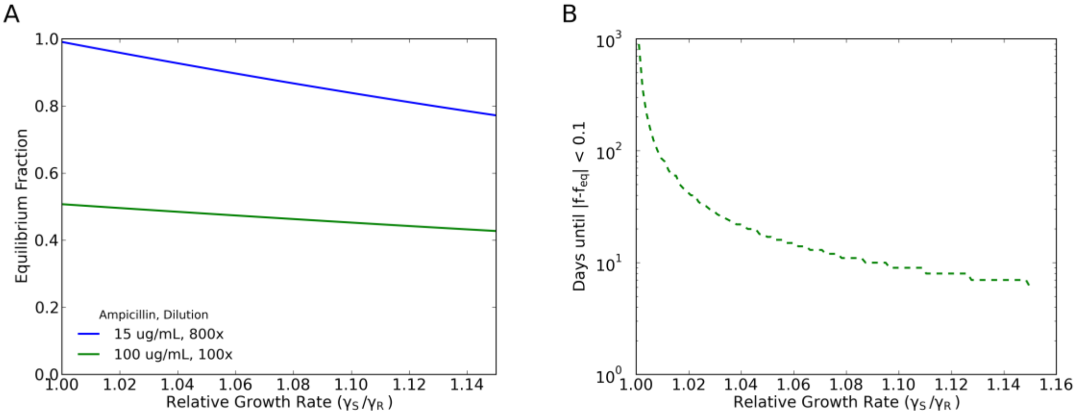

Supplementary Figure 19: Effects of the cost of resistance on the equilibrium fraction and the time to reach equilibrium. (A) The equilibrium fraction depends only weakly on the cost of resistance. For example, if the cost of resistance ($\Delta\gamma=\gamma_S-\gamma_R$) decreases by 50% – which corresponds the relative growth rate ($r=\gamma_S/\gamma_R$) decreasing from 1.15 to 1.07 – the equilibrium fraction increases by ~8% (f_{eq}~0.43 →f_{eq}~0.47) for the 100 µg/mL ampicillin and 100x dilution factor condition. (B) The time to reach equilibrium can change significantly with the cost of resistance. In this simulation, for each relative growth rate, the population was started at a resistant fraction of 0.99, and the time to reach a fraction within 0.1 of the equilibrium was determined. The simulation was carried out for the 100 µg/mL ampicillin and 100x dilution factor condition. As the cost of resistance approaches 0 (r=1), the time to reach equilibrium increases. Note that when the cost of resistance is precisely 0 (r=1), the equilibrium fraction becomes degenerate. In this case, as long as the antibiotic is cleared within the lag time, the fraction of resistant cells does not change since with r=1, the growth rates of resistant and sensitive cells are identical. Parameter values for the simulation are provided in the Supplementary Materials.

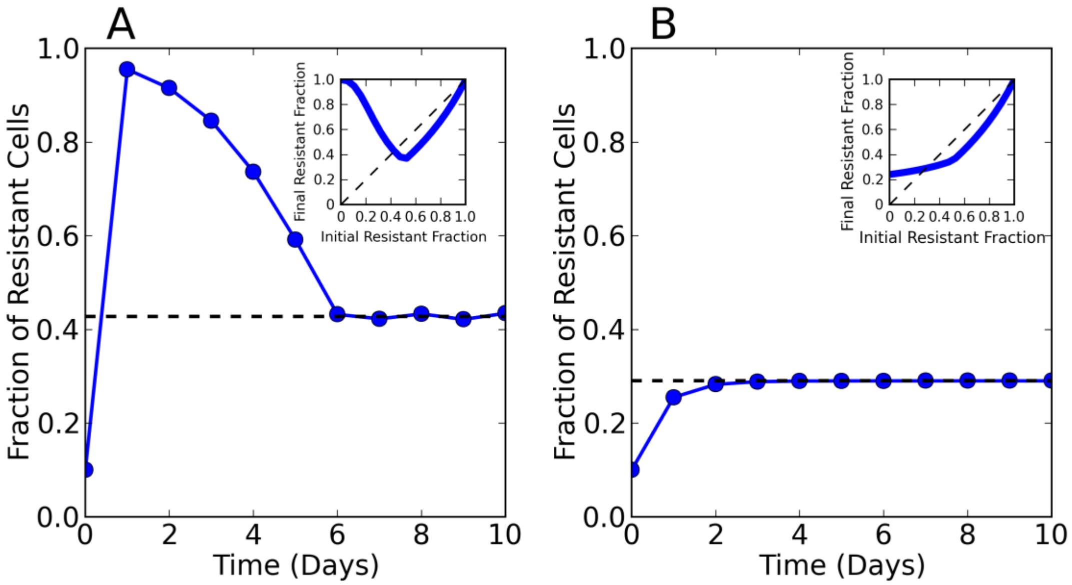

Supplementary Figure 20: Difference between bactericidal and bacteriostatic antibiotics. Time course and difference equation map for the death rate of sensitive cells set to (A) 2.8/hr vs. (B) 0/hr. The plots show that the killing of sensitive cells by the antibiotic is responsible for the overshoot of the fraction above the equilibrium fraction when starting at a low initial fraction of resistant cells and for the "V" of the difference equation maps. This occurs because below the equilibrium fraction, there are not enough resistant cells to inactivate the antibiotic before it starts to affect the growth of sensitive cells. By killing sensitive cells, a bactericidal antibiotic exerts stronger selection for resistant cells than a bacteriostatic antibiotic that just inhibits the growth of sensitive cells. Simulations were run for an antibiotic concentration of 100 μg/mL and a dilution factor of 100x. Other parameter values can be found in the modeling section of the Supplementary Materials.

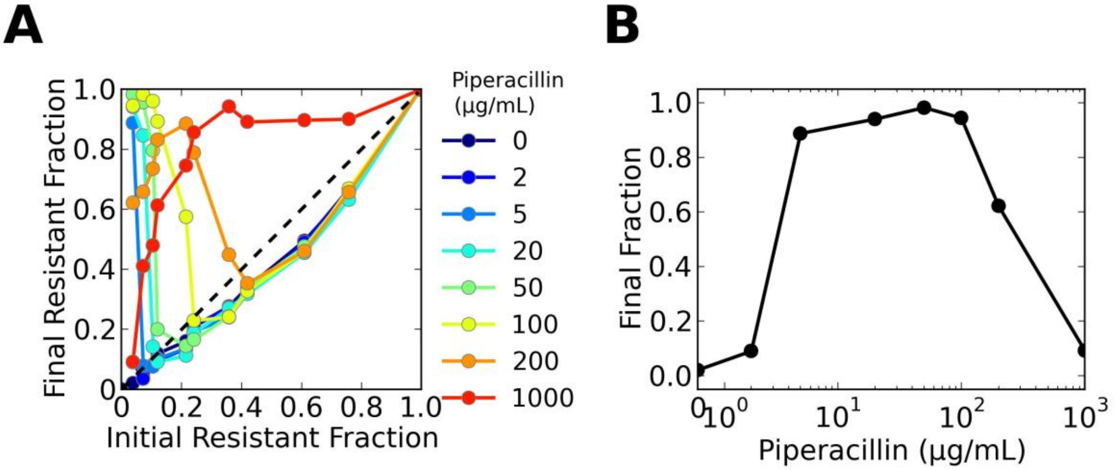

Supplementary Figure 21: Non-monotonic behavior in piperacillin. (A) Difference equation map collected in the antibiotic piperacillin. This difference equation maps behaves in accordance with model expectations at low antibiotic concentrations. However, it deviates from predicted behavior at high concentrations of the antibiotic, in which the difference equation maps start to curve down at low initial fractions of resistant cells. (B) Final fraction of resistant cells as a function of the antibiotic concentration revealing non-monotonic selection for resistance with increasing antibiotic concentrations. The non-monotonic behavior is significant: the final resistant fraction reduces from nearly 1 at intermediate piperacillin concentrations to ~0.1 at high piperacillin concentrations, revealing nearly complete cancelation of selection for resistance at high antibiotic concentrations. For all shown data points, the cultures grew to the same saturation density. Subplot (B) is a cross section through subplot (A) corresponding to an initial fraction of resistant cells at ~4%. Data was acquired at a 100x dilution in an LB medium in the absence of kanamycin.

One possible explanation for the deviation from our model and the non-monotonic behavior is that, at the higher antibiotic concentrations, resistant cells begin to lyse releasing β-

lactamase enzymes into the extra-cellular space (Sykes & Matthew, 1976). Extracellularly these enzymes hydrolyze their antibiotic more quickly (higher substrate concentration), leading to the survival of more sensitive cells.

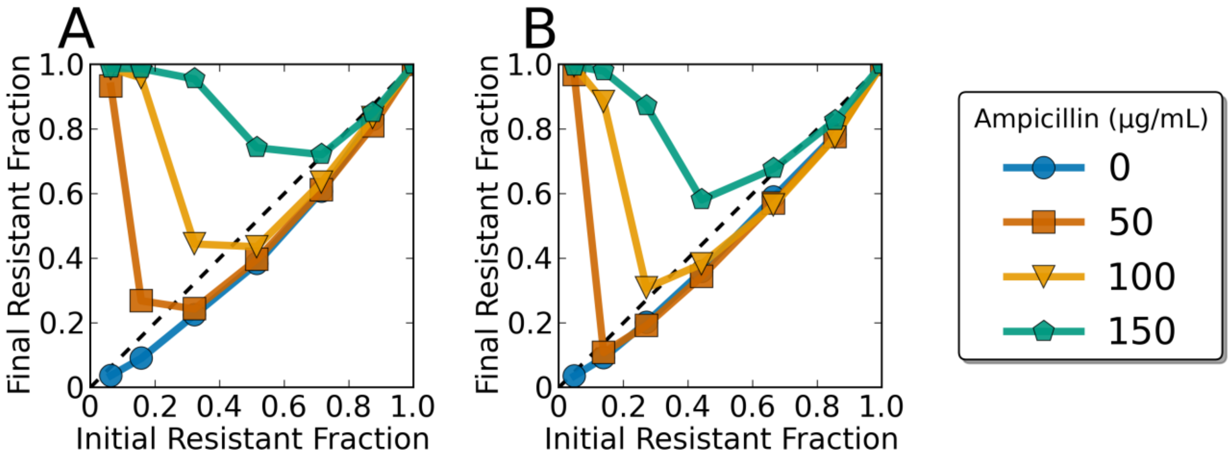

Supplementary Figure 22: Controls showing difference equations obtained with fluorescent markers swapped. The resistant and sensitive strains used in the main text expressed CFP and YFP, respectively. To show that the evolutionary dynamics were independent of any differential cost involved in expressing CFP vs. YFP, we created control strains in which we swapped the fluorescent markers in places. (A) Difference equation obtained with resistant and sensitive strains from the main text. (B) Difference equation obtained with CFP and YFP swapped. Data was acquired in LB with 5 µg/mL of kanamycin at 100x dilution.

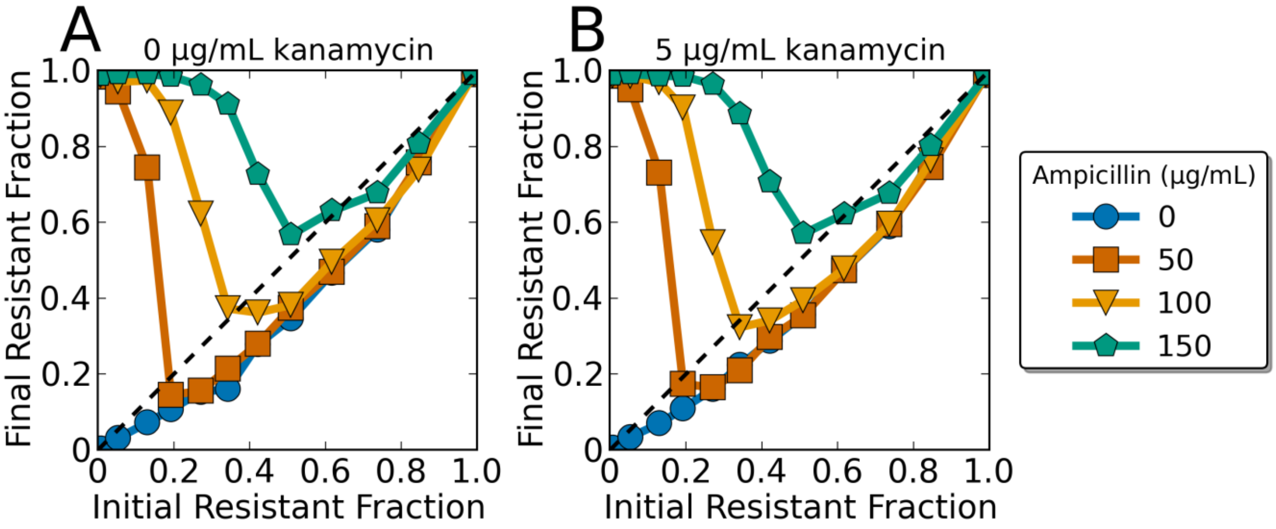

Supplementary Figure 23: Addition of 5 μg/mL kanamycin does not significantly affect the evolutionary dynamics. Both resistant and sensitive cells carry a plasmid that encodes for fluorescence and kanamycin resistance. To make sure that this plasmid was not lost during multi-day experiments, a background concentration of 5 μg/mL of kanamycin was added. Kanamycin was used only as a precaution, and its absence or presence (at 5 μg/mL) did not affect the evolutionary dynamics. Difference equations were mapped for 4 different antibiotic concentrations in (A) no kanamycin or in (B) 5 μg/mL kanamycin. Data was acquired at 100x dilution.

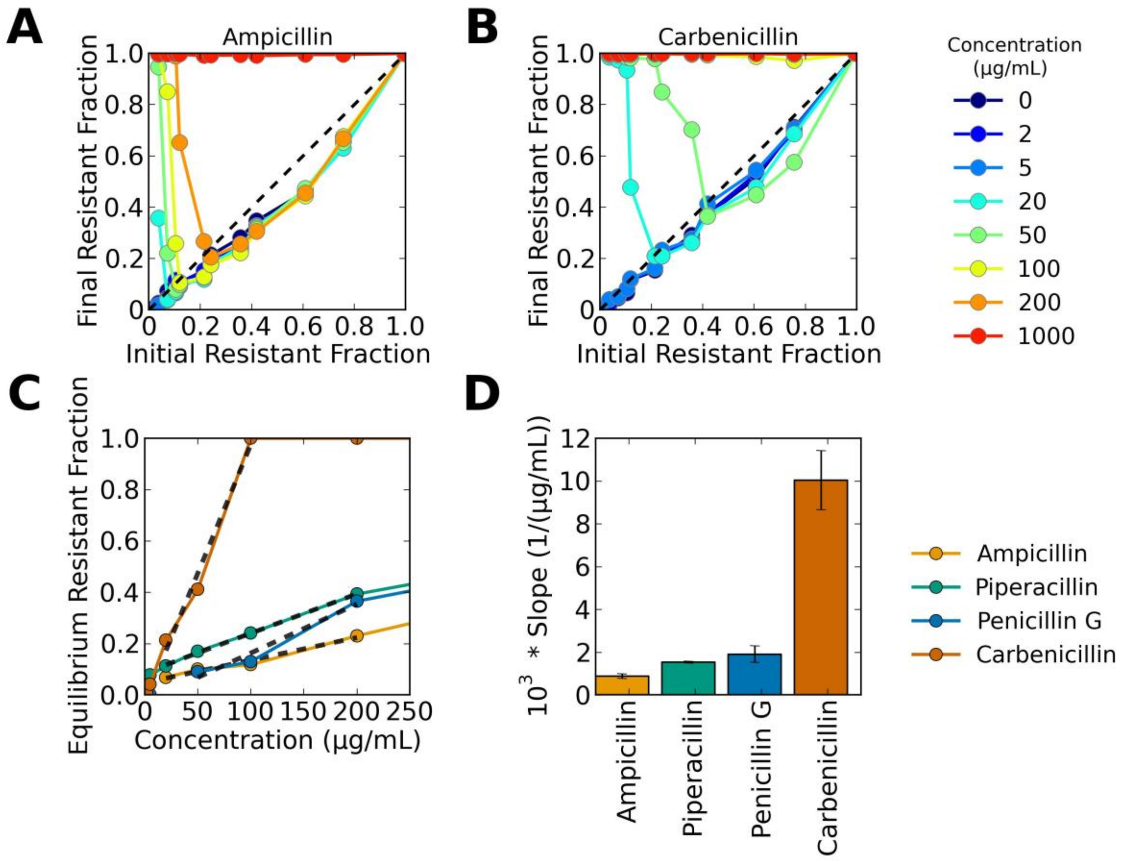

Supplementary Figure 24: Population dynamics in different beta-lactam antibiotics. Examples of difference equation maps collected in the beta-lactam antibiotics (A) ampicillin and (B) carbenicillin. (C) Equilibrium fractions extracted from difference equation maps for the beta-lactam antibiotics ampicillin, piperacillin, penicillin G and carbenicillin. Consistent with model prediction, the equilibrium fractions are approximately linear in the antibiotic concentration. Deviations from linearity can appear at low concentrations due to the non-zero Km of Michaelis-Menten hydrolysis of the antibiotic (see main text for explanation). Black dashes lines represent the range over which a line was fit to the data to calculate the slope. (D) The values of the slopes of the equilibrium fraction vs. the antibiotic concentrations acquired at high antibiotic concentrations. The ratio between these slopes should be proportional to the inverse ratio of the

hydrolysis rates of these antibiotics. TEM-1 hydrolyzes the antibiotics ampicillin, penicillin G, piperacillin and carbenicillin with approximate kcats of 1200/s, 1100/s, 1000/s and 110/s, respectively (Petit *et al*, 1995). Hence, the model would predict that the slope of carbenicillin as compared to the other antibiotics should be about 10 times larger, which is close to experimental observations. Error bars only capture the error from the linear fit.

We note that the equilibrium fractions for ampicillin are significantly different than those presented in the main text (the equilibrium fractions are off by a factor of ~2-3). The data for this figure (and for supplementary figures 8, 16, 18, and 21) was collected more than a year after the data presented everywhere else in the paper. We do not know what has caused this drift, but we have eliminated differences in the antibiotic stock, in strains or in protocol as possible explanations. The drift could be caused by other parameters that we do not normally control for (e.g., humidity, different filter for de-ionized water, batch-to-batch variations in LB). If anything, the equilibrium fractions here show that the sensitive strains can survive in even larger antibiotic concentrations than indicated in the main text.

Data in subplots (A-D) was acquired at a 100x dilution in an LB medium in the absence of kanamycin.

References mentioned in figure captions

Bush K (1988) Beta-lactamase inhibitors from laboratory to clinic. *Clin Microbiol Rev* **1:** 109–123

Foulds G (1986) Pharmacokinetics of Sulbactam/Ampicillin in Humans: A Review. *Clinical Infectious Diseases* **8:** S503–S511

Foulds G, Stankewich JP, Marshall DC, O'Brien MM, Hayes SL, Weidler DJ & McMahon FG (1983) Pharmacokinetics of sulbactam in humans. *Antimicrob. Agents Chemother.* **23:** 692–699

Petit A, Maveyraud L, Lenfant F, Samama JP, Labia R & Masson JM (1995) Multiple substitutions at position 104 of beta-lactamase TEM-1: assessing the role of this residue in substrate specificity. *Biochem J* **305:** 33–40

Sykes RB & Matthew M (1976) The β-lactamases of Gram-negative bacteria and their rôle in resistance to β-lactam antibiotics. *J. Antimicrob. Chemother.* **2:** 115–157

Supporting Information: Modeling Section

Eugene A. Yurtsev, Hui Xiao Chao, Manoshi S. Datta, Tatiana Artemova and Jeff Gore

May 8, 2013

Contents

1 Overview **2**

2 Definition of Parameters **2**

3 Analytic Solutions for the Equilibrium Fraction **3**

4 Summary of Models **4**

4.1 Model 1 4

4.2 Model 4 4

4.3 Model 7 5

5 Sample Derivation of Equilibrium Fractions **5**

5.1 Equilibrium Relations 5

5.2 Solving Model 1 6

5.2.1 Step 1: Solve for the time when the antibiotic concentration drops below the MIC 6

5.2.2 Step 2: Solve for the saturation time 6

5.2.3 Step 3: Solve for the equilibrium fraction 7

5.3 Solving Model 7 7

5.3.1 Step 1: Solve for the time when the antibiotic concentration drops below the MIC 7

5.3.2 Step 2: Solve for the saturation time 8

5.3.3 Step 3: Solve for the equilibrium fraction 8

6 Fitting Experimental Data **9**

6.1 Qualitative Behavior of the Equilibrium Fraction 9

6.2 Parameter Values for Simulations 10

1 Overview

Here, we present a model of bacterial growth in the antibiotic. We show that the way the equilibrium fraction scales with the initial antibiotic concentration, A_i, and cell density, N_i, is independent of many aspects of bacterial growth in the antibiotic. As long as the antibiotic is inactivated according to Michaelis-Menten kinetics, the equilibrium fraction scales approximately as $f_{eq} \propto (A_i + K_M \ln(A_i))/N_i$.

Section (2) defines the parameters used in the models. Section (3) provides a summary of the analytical expressions of the equilibrium fractions for different model variations. Section (4) shows how various aspects of bacterial growth in the antibiotic are incorporated into the models. Two of the models are solved in section (5). Finally, in section (6), we discuss the model used in the main text.

2 Definition of Parameters

Parameter	Definition
γ_R	growth rate of resistant cells
γ_S	growth rate of sensitive cells
γ_D	death rate of sensitive cells
$\Delta\gamma$	$\gamma_S - \gamma_R > 0$
V_{max}	hydrolysis rate
K_M	Michaelis-Menten constant
A_i	initial antibiotic concentration
MIC	antibiotic concentration above which sensitive cells die
ΔA	$A_i - MIC \geq 0$
f_i	initial resistant fraction
f_f	final resistant fraction
f_{eq}	$f_{eq} = f_i = f_f$ is the equilibrium fraction
$N_{Ri} \equiv N_R(0)$	initial number of resistant cells
$N_{Rf} \equiv N_R(\tau_{sat})$	final number of resistant cells
$N_{Si} \equiv N_S(0)$	initial number of sensitive cells
$N_{Sf} \equiv N_S(\tau_{sat})$	final number of sensitive cells
$N_i = N_{Ri} + N_{Si}$	the initial number of both resistant and sensitive cells
$N_{sat} = N_{Rf} + N_{Sf}$	saturation number of both resistant and sensitive cells
τ_{lag}^R	for $t < \tau_{lag}$ resistant cells neither divide nor die
τ_{lag}^S	for $t < \tau_{lag}$ sensitive cells neither divide nor die

Parameter	Definition
τ_{sat}	time at which saturation is reached
τ_b	time at which the antibiotic is broken down

3 Analytic Solutions for the Equilibrium Fraction

#	Lag Phase		Cell Death	Antibiotic Inactivation	f_{eq}
1	✗	$\tau_{lag}^R = \tau_{lag}^S = 0$	✗	$\frac{dA}{dt} = -V_{max}N_{Ri}$	$\frac{\Delta A}{V_{max}N_i \frac{1}{\gamma_R} \ln(\frac{N_{sat}}{N_i})^{\frac{\Delta\gamma}{\gamma_S}}}$
2	✗	$\tau_{lag}^R = \tau_{lag}^S = 0$	✗	$\frac{dA}{dt} = -V_{max}N_R(t)$	$\frac{\Delta A}{V_{max}N_i\{\frac{1}{\gamma_R}[(\frac{N_{sat}}{N_i})^{\frac{\Delta\gamma}{\gamma_S}} - 1]\}}$
3	✗	$\tau_{lag}^R = \tau_{lag}^S = 0$	✗	$\frac{dA}{dt} = -V_{max}N_R(t)\frac{A}{A+K_M}$	$\frac{\Delta A + K_M \ln(\frac{A_i}{MIC})}{V_{max}N_i\{\frac{1}{\gamma_R}[(\frac{N_{sat}}{N_i})^{\frac{\Delta\gamma}{\gamma_S}} - 1]\}}$
4	✗	$\tau_{lag}^R = \tau_{lag}^S = 0$	✓	$\frac{dA}{dt} = -V_{max}N_R(t)$	$\frac{\Delta A}{V_{max}N_i\{\frac{1}{\gamma_R}[(\frac{N_{sat}}{N_i})^{\frac{\Delta\gamma}{\gamma_S+\gamma_D}} - 1]\}}$
5	✓	$\tau_{lag}^R = \tau_{lag}^S \neq 0$	✗	$\frac{dA}{dt} = -V_{max}N_R(t)$	$\frac{\Delta A}{V_{max}N_i\{\tau_{lag}^R + \frac{1}{\gamma_R}[(\frac{N_{sat}}{N_i})^{\frac{\Delta\gamma}{\gamma_S}} - 1]\}}$
6	✓	$\tau_{lag}^R = \tau_{lag}^S \neq 0$	✓	$\frac{dA}{dt} = -V_{max}N_R(t)$	$\frac{\Delta A}{V_{max}N_i\{\tau_{lag} + \frac{1}{\gamma_R}[(\frac{N_{sat}}{N_i})^{\frac{\Delta\gamma}{\gamma_S+\gamma_D}} - 1]\}}$
7	✓	$\tau_{lag}^R = \tau_{lag}^S \neq 0$	✓	$\frac{dA}{dt} = -V_{max}N_R(t)\frac{A}{A+K_M}$	$\frac{\Delta A + K_M \ln(\frac{A_i}{MIC})}{V_{max}N_i\{\tau_{lag} + \frac{1}{\gamma_R}[(\frac{N_{sat}}{N_i})^{\frac{\Delta\gamma}{\gamma_S+\gamma_D}} - 1]\}}$
8	✓	$\tau_{lag}^R \neq \tau_{lag}^S$	✓	$\frac{dA}{dt} = -V_{max}N_R(t)\frac{A}{A+K_M}$	$\frac{\Delta A + K_M \ln(\frac{A_i}{MIC})}{V_{max}N_i\{\tau_{lag}^R + \frac{1}{\gamma_R}[e^{\frac{\gamma_R\gamma_D(\tau_{lag}^S - \tau_{lag}^R)}{\gamma_S+\gamma_D}}(\frac{N_{sat}}{N_i})^{\frac{\Delta\gamma}{\gamma_S+\gamma_D}} - 1]\}}$
9	✓	$\tau_{lag}^R \neq \tau_{lag}^S$	✓	$\frac{dA}{dt} = -V_{max}N_{Ri}\frac{A}{A+K_M}$	$\frac{\Delta A + K_M \ln(\frac{A_i}{MIC})}{V_{max}N_i\{\frac{\gamma_S\tau_{lag}^R + \gamma_D\tau_{lag}^S}{\gamma_S+\gamma_D} + \frac{\Delta\gamma}{\gamma_R(\gamma_S+\gamma_D)}\ln(\frac{N_{sat}}{N_i})\}}$

4 Summary of Models

Here, we explain models 1, 4, and 8 in more detail, writing down the system of differential equations that corresponds to each model. In all the models, we assume that cell growth ceases when the total cell density reaches the saturation density (N_{sat}). Hence, if τ_{sat} corresponds to the time when $N_R + N_S = N_{sat}$, then for $t \geq \tau_{sat}$:

$$\begin{aligned} \frac{dN_R}{dt} &= 0 \\ \frac{dN_S}{dt} &= 0 \end{aligned}$$

4.1 Model 1

Here, resistant cells divide at a characteristic rate of γ_R. Sensitive cells divide at a rate of γ_S when the antibiotic concentration, A, is below their MIC value. When the antibiotic concentration is above their MIC, sensitive cells do not divide. Antibiotic is broken down at constant rate proportional to the initial number of resistant cells ($N_{Ri} = f_R N_i$).

$$\begin{aligned} \frac{dN_R}{dt} &= \gamma_R N_R \\ \frac{dN_S}{dt} &= \begin{cases} \gamma_S N_S & A < MIC \\ 0 & A > MIC \end{cases} \\ \frac{dA}{dt} &= -V_{max} N_{Ri} \end{aligned}$$

4.2 Model 4

Next, we incorporate cell death into the model, saying that for antibiotic concentration above the MIC of sensitive cells, sensitive cells die at a rate $-\gamma_D$. This gives the set of equations:

$$\begin{aligned} \frac{dN_R}{dt} &= \gamma_R N_R \\ \frac{dN_S}{dt} &= \begin{cases} \gamma_S N_S & A < MIC \\ -\gamma_D N_S & A > MIC \end{cases} \\ \frac{dA}{dt} &= -V_{max} N_R \end{aligned}$$

Note that in this example, antibiotic breakdown at time t is proportional to the number of resistant cells at time t (rather than the initial number of resistant cells).

4.3 Model 7

To more realistically model bacterial growth in the antibiotic, we can use Michaelis-Menten Kinetics for antibiotic inactivation; i.e., $\frac{dA}{dt} = -V_{max}N_{Ri}\frac{A}{A+K_M}$. In addition, we can introduce a lag phase during which cells neither grow nor die, giving:

For $t < \tau_{lag}$:

$$\begin{aligned} \frac{dN_R}{dt} &= 0 \\ \frac{dN_S}{dt} &= 0 \\ \frac{dA}{dt} &= -V_{max}N_{Ri}\frac{A}{A+K_M} \end{aligned}$$

For $t \geq \tau_{lag}$:

$$\begin{aligned} \frac{dN_R}{dt} &= \gamma_R N_R \\ \frac{dN_S}{dt} &= \begin{cases} \gamma_S N_S & A < MIC \\ -\gamma_D N_S & A \geq MIC \end{cases} \\ \frac{dA}{dt} &= -V_{max}N_R\frac{A}{A+K_M} \end{aligned}$$

Whether it is necessary to incorporate Michaelis-Menten Kinetics or not depends on the values of K_M and MIC . If $K_M \ll MIC$, then antibiotic breakdown proceeds essentially at saturation for antibiotic concentrations above the MIC.

5 Sample Derivation of Equilibrium Fractions

The models presented thus far describe how bacteria grow in the antibiotic during the course of a single day. These models provide a way to investigate the evolutionary dynamics between resistant and sensitive cells by allowing one to determine how the final resistant fraction, f_f, after 23 hours of growth depends on the initial resistant fraction, f_i, antibiotic concentration, A_i, and cell density, N_i. It is straightforward to integrate these models numerically to find the required dependence; however, it is challenging to gain intuition from numerical solutions. Fortunately, we can obtain an exact analytical expression for the dependence of equilibrium resistant fraction ($f_i = f_f = f_{eq}$) on the initial antibiotic concentration and cell density.

We will first derive two useful relations between the initial and final cell densities that hold at the equilibrium fraction, and then proceed to use these relations to derive an analytical expression for the equilibrium fraction.

5.1 Equilibrium Relations

At equilibrium, $f_f = f_i$. This is equivalent to saying that:

$$\frac{N_{Rf}}{N_{sat}} = \frac{N_{Ri}}{N_i}$$

Therefore,

$$\boxed{\frac{N_{Rf}}{N_{Ri}} = \frac{N_{sat}}{N_i}} \tag{5.1}$$

If we use the same logic as above but start from the expression$1 - f_f = 1 - f_i$, we get:

$$\boxed{\frac{N_{Sf}}{N_{Si}} = \frac{N_{sat}}{N_i}} \tag{5.2}$$

5.2 Solving Model 1

Model 1 (see 4.1) ignores lag and cell death, and it assumes that antibiotic hydrolysis proceeds at a constant rate proportional to the initial cell density of resistant cells. Due to its simplicity, we can solve this model without doing much algebra.

5.2.1 Step 1: Solve for the time when the antibiotic concentration drops below the MIC

$$\frac{dA}{dt} = -V_{max}N_{Ri}$$

$$\frac{\Delta A}{\tau_b} = V_{max}N_{Ri}$$

$$\boxed{\tau_b = \frac{\Delta A}{V_{max}N_{Ri}}}$$

5.2.2 Step 2: Solve for the saturation time

Let's start with the equation (5.1):

$$\frac{N_{Rf}}{N_{Ri}} = \frac{N_{sat}}{N_i}$$

For this simple model, the number of resistant cells increases with time according to the equation: $N_{Rf} = N_{Ri}e^{\gamma_R \tau_{sat}}$; therefore, $\frac{N_{Rf}}{N_{Ri}} = e^{\gamma_R \tau_{sat}}$, so:

$$e^{\gamma_R \tau_{sat}} = \frac{N_{sat}}{N_i},$$

giving:

$$\boxed{\tau_{sat} = \frac{1}{\gamma_R} ln(\frac{N_{sat}}{N_i})}$$

5.2.3 Step 3: Solve for the equilibrium fraction

$$\frac{N_{Sf}}{N_{Si}} = \frac{N_{sat}}{N_i} \ (5.2)$$

Since the sensitive cells do not start growing until the antibiotic is broken down, the number of sensitive cells increases with time according to the equation: $N_{Sf} = N_{Si}e^{\gamma_S(\tau_{sat}-\tau_b)}$; therefore, $\frac{N_{Sf}}{N_{Si}} = e^{\gamma_S(\tau_{sat}-\tau_b)}$. Combining this with the previous expression, we get:

$$e^{\gamma_S(\tau_{sat}-\tau_b)} = \frac{N_{sat}}{N_i}$$

$$\tau_{sat} - \tau_b = \frac{1}{\gamma_S}\ln(\frac{N_{sat}}{N_i})$$

Next, we substitute in the expressions we got for τ_{sat} and τ_b:

$$\frac{1}{\gamma_R}ln(\frac{N_{sat}}{N_0}) - \frac{\Delta A}{V_{max}N_{Ri}} = \frac{1}{\gamma_S}\ln(\frac{N_{sat}}{N_i})$$

$$\frac{\Delta A}{V_{max}N_{Ri}} = (\frac{1}{\gamma_R} - \frac{1}{\gamma_S})ln(\frac{N_{sat}}{N_i})$$

$$\frac{\Delta A}{V_{max}N_i f_{eq}} = \frac{1}{\gamma_R}ln(\frac{N_{sat}}{N_i})^{\frac{\Delta\gamma}{\gamma_S}}$$

$$\boxed{f_{eq} = \frac{\Delta A}{V_{max}N_i \frac{1}{\gamma_R} ln(\frac{N_{sat}}{N_i})^{\frac{\Delta\gamma}{\gamma_S}}}}$$

5.3 Solving Model 7

Model 7 (see 4.3) incorporates a few more features, so the algebra is a bit messier, but the same general procedure applies.

5.3.1 Step 1: Solve for the time when the antibiotic concentration drops below the MIC

The antibiotic is inactivated according to Michaelis-Menten kinetics:

$$\frac{dA}{dt} = -V_{max}N_R\frac{A}{A+K_M},$$

where $N_R(t < \tau_{lag}) = N_{Ri}$ and $N_R(\tau_{lag} < t < \tau_{sat}) = N_{Ri}e^{\gamma_R(t-\tau_{sat})}$. We need to solve this differential equation to find the time, τ_b, at which the antibiotic concentration drops below the MIC of sensitive cells.

$$\frac{A+K_M}{A}dA = -V_{max}N_R dt$$

$$\int(1+\frac{K_M}{A})dA = -\int V_{max}N_R dt$$

$$(A + K_M\ln(A))|_{A_i}^{MIC} = -\int_0^{\tau_b} V_{max}N_R dt$$

$$-\Delta A + K_M \ln(\frac{MIC}{A_i}) = -V_{max} N_{Ri}(\tau_{lag} + \frac{1}{\gamma_R} e^{\gamma_R(t-\tau_{lag})}|_{\tau_{lag}}^{\tau_b})$$

$$\Delta A + K_M \ln(\frac{A_i}{MIC}) = V_{max} N_{Ri}(\tau_{lag} + \frac{1}{\gamma_R}(e^{\gamma_R(\tau_b-\tau_{lag})} - 1))$$

$$\frac{\Delta A + K_M \ln(\frac{A_i}{MIC})}{V_{max} N_{Ri}} = \tau_{lag} + \frac{1}{\gamma_R}(e^{\gamma_R(\tau_b-\tau_{lag})} - 1)$$

Let's introduce $\tau_b^* = \tau_b^0 + \tau_b^1$, where $\tau_b^0 = \frac{\Delta A}{V_{max} N_{Ri}}$ and $\tau_b^1 = \frac{K_M \ln(\frac{A_i}{MIC})}{V_{max} N_{Ri}}$. Then,

$$\tau_b^* = \tau_{lag} + \frac{1}{\gamma_R}(e^{\gamma_R(\tau_b-\tau_{lag})} - 1)$$

$$\frac{1}{\gamma_R}(e^{\gamma_R(\tau_b-\tau_{lag})} - 1) = \tau_b^* - \tau_{lag}$$

$$e^{\gamma_R(\tau_b-\tau_{lag})} - 1 = \gamma_R(\tau_b^* - \tau_{lag})$$

$$\gamma_R(\tau_b - \tau_{lag}) = \ln(1 + \gamma_R(\tau_b^* - \tau_{lag}))$$

$$\boxed{\tau_b = \tau_{lag} + \frac{1}{\gamma_R}\ln(1 + \gamma_R(\tau_b^* - \tau_{lag}))}$$

5.3.2 Step 2: Solve for the saturation time

$$\frac{N_{Rf}}{N_{Ri}} = \frac{N_{sat}}{N_i} \quad (5.1)$$

$$e^{\gamma_R(\tau_{sat}-\tau_{lag})} = \frac{N_{sat}}{N_i}$$

$$\gamma_R(\tau_{sat} - \tau_{lag}) = \ln(\frac{N_{sat}}{N_i})$$

$$\boxed{\tau_{sat} = \tau_{lag} + \frac{1}{\gamma_R}\ln(\frac{N_{sat}}{N_i})}$$

5.3.3 Step 3: Solve for the equilibrium fraction

$$\frac{N_{Sf}}{N_{Si}} = \frac{N_{sat}}{N_i} \quad (5.2)$$

$$e^{\gamma_S(\tau_{sat}-\tau_b)-\gamma_D(\tau_b-\tau_{lag})} = \frac{N_{sat}}{N_i}$$

$$\gamma_S(\tau_{sat} - \tau_b) - \gamma_D(\tau_b - \tau_{lag}) = \ln(\frac{N_{sat}}{N_i})$$

$$\gamma_S \tau_{sat} + \gamma_D \tau_{lag} - (\gamma_S + \gamma_D)\tau_b = \ln(\frac{N_{sat}}{N_i})$$

$$\gamma_S \tau_{lag} + \frac{\gamma_S}{\gamma_R}\ln(\frac{N_{sat}}{N_i}) + \gamma_D \tau_{lag} - (\gamma_S + \gamma_D)\tau_b = \ln(\frac{N_{sat}}{N_i})$$

$$(\gamma_S+\gamma_D)\tau_{lag}-(\gamma_S+\gamma_D)\tau_b=(1-\frac{\gamma_S}{\gamma_R})\ln(\frac{N_{sat}}{N_i})$$

$$(\gamma_S+\gamma_D)\tau_{lag}-(\gamma_S+\gamma_D)(\tau_{lag}+\frac{1}{\gamma_R}\ln(1+\gamma_R(\tau_b^*-\tau_{lag})))=(1-\frac{\gamma_S}{\gamma_R})\ln(\frac{N_{sat}}{N_i})$$

$$-\ln(1+\gamma_R(\tau_b^*-\tau_{lag}))=\frac{\gamma_R-\gamma_S}{\gamma_S+\gamma_D}\ln(\frac{N_{sat}}{N_i})$$

$$1+\gamma_R(\tau_b^*-\tau_{lag})=(\frac{N_{sat}}{N_i})^{-\frac{\gamma_R-\gamma_S}{\gamma_S+\gamma_D}}$$

$$\gamma_R\tau_b^*=\gamma_R\tau_{lag}+(\frac{N_{sat}}{N_i})^{\frac{\gamma_S-\gamma_R}{\gamma_S+\gamma_D}}-1$$

$$\gamma_R\frac{\Delta A+K_M\ln(\frac{A_i}{MIC})}{V_{max}N_{Ri}}=\gamma_R\tau_{lag}+(\frac{N_{sat}}{N_i})^{\frac{\gamma_S-\gamma_R}{\gamma_S+\gamma_D}}-1$$

$$\boxed{f_{eq}=\frac{\Delta A+K_M\ln(\frac{A_i}{MIC})}{V_{max}N_i\{\tau_{lag}+\frac{1}{\gamma_R}[(\frac{N_{sat}}{N_i})^{\frac{\gamma_S-\gamma_R}{\gamma_S+\gamma_D}}-1]\}}} \tag{5.3}$$

6 Fitting Experimental Data

To fit the experimental data, we used model #7 which incorporates cell death, Michaelis-Menten hydrolysis and lag time (see section 4.3). We fit the equilibrium fractions (Figure 3C) to the full analytical solution of this model (Eq. 5.3), acquiring estimates for the parameters MIC, V_{max}, K_M.

We then plugged the values of MIC, V_{max}, K_M into the differential equations describing model #7 (see section 4.3) and integrated the differential equations numerically to recapitulate the difference equation maps (Figure 3A, B).

To fit the data with the tazobactam inhibitor (Figure 4C), we used equation 5.3, modifying $K_M \to K_{eff} = K_M \cdot (1+[I]/K_I)$. We fit the data using a single free parameter (K_i). (Other parameter values were held fixed. The values of MIC, V_{max} and K_m were set to those acquired in the fit of Figure 3C.)

6.1 Qualitative Behavior of the Equilibrium Fraction

The analytical expression of the equilibrium fraction is (Eq. 5.3):

$$f_{eq}=\frac{\Delta A+K_M\ln(\frac{A_i}{MIC})}{V_{max}N_i\{\tau_{lag}+\frac{1}{\gamma_R}[(\frac{N_{sat}}{N_i})^{\frac{\Delta\gamma}{\gamma_S+\gamma_D}}-1]\}}$$

Because in our experiments N_i varies by less than an order of magnitude and $\frac{\Delta\gamma}{\gamma_S+\gamma_D} \ll 1$, the equilibrium fraction can be approximated by the expression:

$$f_{eq}\approx C\frac{\Delta A+K_M\ln(\frac{A_i}{MIC})}{V_{max}N_i}, \tag{6.1}$$

where $C=\frac{1}{\tau_{lag}+\frac{1}{\gamma_R}[(\frac{N_{sat}}{N_i})^{\frac{\Delta\gamma}{\gamma_S+\gamma_D}}-1]}$.

Using the numbers in section (6.2), C works out to be ≈ 0.84 hr^{-1} for a dilution factor of 100x, and ≈ 0.78 hr^{-1} for a dilution factor of 800x. This amounts to less than a 10% change in C, which means that C can be treated essentially as a constant. With C a constant, equation 6.1 implies that the equilibrium fraction (f_{eq}) scales approximately inversly with the initial cell density (N_i).

Furthermore, for initial antibiotic concentrations higher than the dissociation constant ($A_i \gg K_M$), the contribution of the logarithmic term becomes small and the expression simplifies to:

$$f_{eq} \approx C \frac{\Delta A}{V_{max} N_i}$$

This relationship indicates that plotting the number of resistant cells at equilibrium (f_{Ri}) vs. the initial antibiotic concentration (A_i) should yield a line with a slope of ($\frac{C}{V_{max}}$):

$$\boxed{f_{Ri} = f_{eq} \times N_i \approx \frac{C}{V_{max}} \Delta A = \frac{C}{V_{max}} A_i - \frac{C}{V_{max}} MIC}$$

6.2 Parameter Values for Simulations

The following table lists the parameters used for simulations (see supplementary figures for details):

Parameter	Meaning	Value	Source
γ_R	growth rate of resistant cells	$1.1\,\text{hr}^{-1}$	measured experimentally
$\frac{\gamma_S}{\gamma_R}$	relative fitness of sensitive cells	1.15	measured experimentally
γ_D	death rate of sensitive cells	$2.8\,\text{hr}^{-1}$	measured experimentally
τ_{lag}	lag time	$\sim 1\,\text{hr}$	measured experimentally[1]
N_{sat}	cell density at saturation (after 23 hours of growth)	$1.2 \times 10^7 \frac{cells}{\mu L}$	measured experimentally
V_{max}	hydrolysis rate	$\sim 10^6 \frac{molecules}{cell \times second}$	determined from fit
K_M	Michaelis-Menten constant for ampicillin inactivation	$6.7 \frac{\mu g}{mL}$	determined from fit
K_I	dissociation constant for tazobactam-enzyme complex	$4.56 \frac{ng}{mL}$	determined from fit
MIC	minimum inhibitory concentration of sensitive cells	$\sim 1.1 \frac{\mu g}{mL}$	determined from fit

[1]The lag time shows some variability. For simplicity, we took it to be 1 hour. Changing the value of the lag time mainly changes the fit value for V_{max}, but it hardly affects the dynamics.
